# Development of a multiplex microsatellite marker set for the study of the solitary red mason bee, *Osmia bicornis* (Megachilidae)

**DOI:** 10.1007/s11033-021-06796-x

**Published:** 2021-11-01

**Authors:** Jens Van Eeckhoven, Gavin J. Horsburgh, Deborah A. Dawson, Kathryn Mayer, Amanda Bretman, Elizabeth J. Duncan

**Affiliations:** 1 School of Biology, University of Leeds, Leeds, LS2 9JT UK; 2 NERC Biomolecular Analysis Facility, Department of Animal and Plant Sciences, University of Sheffield, Sheffield, S10 2TN UK

**Keywords:** Megachilidae, Red mason bee, *Osmia bicornis*, Microsatellite marker, Population structure, Conservation genetics

## Abstract

**Background:**

Solitary bees, such as the red mason bee (*Osmia bicornis*), provide important ecosystem services including pollination. In the face of global declines of pollinator abundance, such haplodiploid Hymenopterans have a compounded extinction risk due to the potential for limited genetic diversity. In order to assess the genetic diversity of *Osmia bicornis* populations, we developed microsatellite markers and characterised them in two populations.

**Methods and results:**

Microsatellite sequences were mined from the recently published *Osmia bicornis* genome, which was assembled from DNA extracted from a single male bee originating from the United Kingdom. Sequences were identified that contained dinucleotide, trinucleotide, and tetranucleotide repeat regions. Seventeen polymorphic microsatellite markers were designed and tested, sixteen of which were developed into four multiplex PCR sets to facilitate cheap, fast and efficient genotyping and were characterised in unrelated females from Germany (n = 19) and England (n = 14).

**Conclusions:**

The microsatellite markers are highly informative, with a combined exclusion probability of 0.997 (first parent), which will enable studies of genetic structure and diversity to inform conservation efforts in this bee.

**Supplementary Information:**

The online version contains supplementary material available at 10.1007/s11033-021-06796-x.

## Introduction

Bee species are experiencing global declines, which is of great concern as they are indispensable pollinators [1–3]. The importance of genetics and genomics to bee conservation is becoming increasingly recognised [1–3]. The risk of extinction can be an order of magnitude higher for bees relative to their diplodiploid counterparts [2, 3]. The increased risk of extinction stems from two compounding effects. (a) Haplodiploid bees are expected to have a 25% reduction in genetic diversity on average, compared to diplodiploid counterparts [2]. This in addition to (b) complementary sex determining systems that can give rise to sterile or inviable diploid males, which further reduce the breeding effective population size [2, 3]. Consequently, ascertaining genetic structure and genetic diversity of hymenopteran pollinators, alongside their sex determining system, will be critical to conservation efforts [1–3].

In order to conserve the valuable ecosystem service of insect pollination in crop production, it could suffice to focus on common or dominant species [4]. Members of the solitary bee genus *Osmia* are considered such dominant crop pollinators, with six members of *Osmia* belonging to the top 100 of bee species with the highest mean contribution to crop production value (*Osmia cornifrons*, *Osmia lignaria*, *Osmia taurus*, *Osmia bicornis*, *Osmia pumila*, and *Osmia virga*; [4]). In fact, *Osmia* have been extensively studied with regard to their potential for crop pollination in green houses and fruit crops [5], and *Osmia bicornis* is already commercially available for this purpose in parts of Asia, Europe and North America. Acquiring genetic information on such a common and dominant species may not only aid conservation efforts, but will also help inform commercial breeding and export practices.

Microsatellite markers are part of the molecular toolbox that can help inform these efforts, particularly in analyses where many individuals are included and subpopulations are of interest [6]. Neumann and Seidelmann [7] have previously identified and validated six microsatellite markers for *O. bicornis*; five dinucleotide repeats and one trinucleotide repeat (Table 1). However, dinucleotide repeats are among the least reliable as they are more prone to stutter and slippage [8]. The resulting shadow bands make dinucleotide repeats harder to call, increasing human error in genotype calling, which can have far reaching consequences [9]. As power of inference relies on the number and variability of loci, and the number of individuals sampled [10], the use of the six existing microsatellites requires large sample sizes to study population genetic structure in *O. bicornis* [11]. The recently sequenced and assembled *O. bicornis* genome [12] using DNA isolated from a male bee sampled in the United Kingdom (Penmenner Rd, Lizard, Helston TR12 7NR in 2015; biosample: SAMN05967202) provides an opportunity to expand on these existing microsatellite markers. Here we identify seventeen new polymorphic microsatellite markers for *O. bicornis* mined from its genome [12], sixteen of which were designed and tested as multiplexes to allow for rapid and cost-effective genotyping of this species.

**Table 1:** Primer sequences of one monomorphic and seventeen polymorphic *Osmia bicornis* microsatellite loci (Obic) mined from the genome [12], as well as the primer sequences of the six existing *Osmia bicornis* markers (Oru) from Neumann and Seidelmann ([7] provided for completeness and ease of access)

Locus	FL	Primer sequence (5ʹ–3 ʹ )	*T*_*m*_	MS	Motif	References
Obic113	6-FAM	F: CTGCCCTCTCGTCTCTTCC	60.08	C	(CCAG)_7_	NW_021683655
		R: AATTCGGGTTGAAACCTGTG	59.83			*3,249,555–3,249,781*
Obic1176	HEX	F: ACGCTTGTCGCTTTCAG	60.14	B	(TGTA)_8_	NW_021683667
		R: TTCTCGAACAGATGTCCTTGG	60.24			*1,318,406–1,318,636*
Obic1181	NED	F: CTCGGGAATCCACCTTATTG	59.38	A	(CTTT)_13_	NW_021683667
		R: TGCCTAGCGAAAGAGGGTAG	59.61			*1,330,169–1,330,419*
Obic1206	HEX	F: CCAACCTTCCCACACCTAAC	59.3	D	(ACCT)_9_	NW_021683667
		R: AACAGGACAAAGGAGCGAAG	59.47			*1,448,980–1,449,214*
Obic1238	6-FAM	F: ACAATTTGTAGGGTGGACACG	59.77	B	(AGCA)_13_	NW_021683667
		R: GCGATTCAACCTCCTTTCAC	59.68			*268,439–268,685*
Obic1252	6-FAM	F: CCTTCCTATGTCGCTGCTG	59.56	C	(TTTC)_17_	NW_021683667
		R: TCCAAGTTCCTGTACCAATGTG	59.89			*362,230–362,496*
Obic1374	6-FAM	F:CTATCCGGCACTCTTTCTCG	59.97	A	(GTTC)_9_	NW_021683668
		R: AAACGCGGAATGAGATATGC	60.07			*896,441–896,675*
Obic168	HEX	F: AGCCACGTTGAAGTTGTTGC	61.28	A	(TTC)_10_	NW_021683656
		R: GGGTTTCTCCGTTCTGCTG	60.79			*1,147,308–1,147,536*
Obic1	HEX	F: CGGTTTATGGCAGGTAAACG	60.37	D	(AG)_14_	NW_021683655
		R: GTAGCAGCAGCCGGTGTATC	60.83			*1,045,524–1,045,750*
Obic220	NED	F: CTGCATCACCTACGCAACTG	60.47	D	(CGCA)_8_	NW_021683656
		R: AACGCGCCAAGTAGAATCTG	60.41			*2,567,542–2,567,772*
Obic415	6-FAM	F: GAATGGGCAACGTCTATTTACAG	59.91	A	(CAGA)_8_	NW_021683658
		R: ATCCTTTGTTGCCGTTTGTC	59.98			*562,062–562,292*
Obic450^a^	6-FAM	F: TTGCCTTTCGAAATCAAGC	58.98	^–^	(GAAG)_6_	NW_021683659
		R: CGACAGATCGAAACGTCATC	59.25			*140,411–140,633*
Obic629	HEX	F: CTGCTTCGGCCTCTTTCTAC	59.22	B	(CTTT)_12_	NW_021683660
		R: AAGTCGGTTCTTCGCATACC	59.2			*1,912,638–1,912,876*
Obic73	HEX	F: CCAATACCTCCCTCTTCTCCTC	60.44	C	(TCC)_14_	NW_021683655
		R: CCCACGTTCTGCCATTACTC	60.52			*2,545,096–2,545,330*
Obic740	NED	F: AGTACGCGTCACGACAAAGAG	60.5	C	(AAGG)_17_	NW_021683661
		R: GTACAACCGGCCATCGTATC	60.22			*26,681–26,947*
Obic77	NED	F: GATCTCGTGTTCACGGTAGG	58.16	B	(GT)_19_	NW_021683655
		R: CTGCAGTTTCCTGGATCG	57.82			*2,568,116–2,568,352*
Obic95	6-FAM	F: TTTAAGGAAACAGCCAGCAG	58.17	D	(GGAA)_9_	NW_021683655
		R: TTCATGAAGTATAAGAGGAAACGAC	58.00			*2,886,250–2,886,484*
Obic428^a^	6-FAM	F: GGGTAAAGGGTTAGGGAACTG	58.88	^–^	(TGGC)_6_	AJ884679
		R: AGCAAGGGTGGTAGTGAAGG	59.21			
Oru10^b^	^–^	F: TTTCATGTTCCGTATTGTCA	50	^–^	(AC)_11_	AJ884680
		R: TGTTCGCTTCCAAAATCA	50			
OruS4^b^	^–^	F: GAACGAAACACCACTGTCTT	50	^–^	(AC)_10_	AJ884681
		R: CACGGCGAGACGAAT	50			
OruE5^b^	^–^	F: CGGAGACTTGGTTGAAAAT	50	^–^	(GA)_13_	AJ884682
		R: AAGCACTACCACCTTTCTTTA	50			
OruS8^b^	^–^	F: TTGGAAAAGAAGCGGATGAG	51	^–^	(AG)_14_	AJ884683
		R: CACCCTCGGAACCACTCTC	51			
OruC4^b^	^–^	F: CGTAGAAAACGAACCCTGTAA	52	^–^	(CT)_13_	AJ884684
		R: CGATAGCCGTATGGTAGCAC	52			
OruA8^b^	^–^	F: TCGCGATGTATCGTGTTCCTT	54	–	(GAA)_9_	AJ884679
		R: GGCTGGCGGCTGTCTAAG	54			

^a^Obic428 was monomorphic, and Obic450 was dimorphic, hence these loci were not tested in multiplex

^b^The existing microsatellite markers from Neumann and Seidelmann [7] were not tested or incorporated here

T_m_ = melting temperature in °C, MS = multiplex primer set (all PCR amplified at 57 °C), FL = fluorophore label, Motif = repeat motif, and Reference = NCBI Reference Sequence ([12]; with genomic location in italics) or EMBL accession number in the case of the Oru markers from [7]

## Materials and methods

Microsatellites were mined from the *O. bicornis* genome [12; Accession Nr. SRP065762] using MISA (MIcroSAtellite; [13]). Di-, tri-, and tetra-nucleotide repeats were mined, with a minimum of eight repeats. Tetranucleotide repeats were preferred as they show less stutter and slippage [8] and are easier to call. Twenty sequences were selected, avoiding poly(N) sequences and composite repeats. Primers were designed in the sequence flanking the repeat regions (20–50 bp away from the repeat) using Primer3 [v 0.4.0; 14] with: the optimum melting temperature (T_m_) set at 60 °C, product size ranging from 100 to 300 bp, a maximum difference in T_m_ of 0.5 °C between forward and reverse primers, a maximum poly(N) of three, a CG clamp, and using Schildkraut and Lifson’s [15] original salt correction formula. These thresholds and conditions were relaxed only when no appropriate primers could be found.

Live *O. bicornis* were obtained from a commercial breeder (Dr Schubert Plant Breeding Landsberg, Germany) from two breeding sites 100 km apart. Additional *O. bicornis* were obtained from MasonBees Ltd. (Shropshire, UK), originating from sites in North Shropshire and Surrey (240 km apart). The commercially bred German sites were treated as being part of the same population, whereas the English sites were treated separately. All individuals were delivered as live cocoons within intact nest tubes as part of a larger study. 45 nest tubes containing 2–16 individuals (mean ± SE = 8.850 ± 1.064) were acquired in total. Adults were sexed, with males having a white tuft on the frons, whereas females possess two horns. As all of the individuals in a single nest tube are presumed to be either siblings or half siblings, a single female was taken from each nest tube (N = 41).

DNA was extracted using hot sodium hydroxide and pH was adjusted using Tris–HCl [HotSHOT; 16]. Specimens were frozen at − 20 °C for 1 day, after which a single leg of each female was removed using sterile tweezers. Legs were placed in a 0.2 ml microcentrifuge tube (Applied Biosystems) and 75 μl of HotSHOT alkaline lysis buffer (25 mM NaOH, 0.2 M EDTA, pH 12) was added. Samples were incubated at 95 °C for 30 min and cooled to 4 °C for 3 min. 75 μl of HotSHOT neutralisation buffer (40 mM Tris–HCl, pH 5) was added to neutralise the pH. Samples were stored at − 20 °C and used within 3 months. Amplification was conducted in 2 μl PCR-reactions following Kenta et al*.* [17]. 0.5–20 ng of DNA template was air-dried at 50 °C for 30 min. 2 μl PCR-reactions were prepared, containing: 1× Multiplex PCR Master Mix (QIAGEN) and 0.2 μM primer mix—containing fluorophore-labelled forward primer (6-FAM and HEX, Sigma-Aldrich; NED, ThermoFisher Scientific) and unlabelled reverse primer in low TE. The PCR profile initiated at 95 °C for 15 min, followed by 45 cycles of 95 °C for 30 s, 57 °C for 1.5 min and 72 °C for 1.5 min. Final extension was performed at 60 °C for 30 min. All PCR reactions were performed with the annealing temperature of 57 °C, as primer sets were designed for the purposes of multiplexing and 57 °C was sufficiently low to amplify all primer sets (Table 1). An ABI 3730 48-well capillary DNA Analyser (ThermoFisher Scientific) was used for genotyping, using GeneScan 500 ROX dye Size Standard (Applied Biosystems). Genotype calling was performed using GeneMapper (v 3.7; Applied Biosystems), with manual binning and scoring of alleles. Alleles were considered polymorphic when the minor allele frequency was larger 0.05.

Individuals need to be less related than half-sibs to correctly test for both Hardy–Weinberg and linkage equilibrium. Using seventeen markers, all females were tested for possible sibship within each population using MLrelate [v 1.0; 18]. A total of eight females were identified as possibly belonging to half-sib pairs, and one female was subsequently removed from each putative half-sib pair for analysis. This left 33 females to be analysed. The German individuals were pooled for analysis, as a previous study indicated that isolation by distance may be both weak and insignificant in this species [11]. The respective sample sizes of each population can be found in Table 2. Allele frequencies, null allele frequencies, and expected and observed heterozygosity were estimated using Cervus [v 3.0.7; 19]; Hardy–Weinberg equilibrium and linkage disequilibrium were tested using GENEPOP [v 4.7; 20,21]. Tests for Hardy–Weinberg equilibrium and linkage disequilibrium were carried out by population, and corrected using false discovery rate [22]. Genotyping data used are given in Online Resource 1. Four multiplexes (Table 1) were designed using Multiplex Manager [23] and AutoDimer [24], and subsequently checked for allelic dropout and non-specific amplicons by comparing replicates of the samples carried out in simplex, duplex and the eventual multiplexes shown in Table 1.

**Table 2:** Characterisation of the seventeen multiplexed *Osmia bicornis* microsatellite loci for three populations

Locus	North Shropshire, England (N = 7)					Surrey, England (N = 7)					Landsberg, Germany (N = 19)					
	*N*_*A*_	*H*_*O*_	*H*_*E*_	*P*_*HW*_	P_SA_	*N*_*A*_	*H*_*O*_	*H*_*E*_	*P*_*HW*_	P_SA_	*N*_*A*_	*H*_*O*_	*H*_*E*_	*P*_*HW*_	Null	P_SA_
**Obic113**	3	0.29	0.28	1	1	5	**0.29**	**0.66**	0.096	1	6	0.42	0.49	0.467	**0.114**	1
Obic1176	7	0.71	0.88	1	1	5	0.71	0.85	1	1	6	0.74	0.74	0.467	− 0.024	1
Obic1181	4	0.71	0.65	1	1	4	0.57	0.71	1	1	8	0.68	0.78	0.716	0.063	1
Obic1206	4	0.86	0.7	1	1	2	0.29	0.26	1	1	6	0.63	0.69	0.737	0.042	1
Obic1238	4	0.57	0.65	0.94	1	4	0.71	0.66	1	1	6	0.79	0.74	0.971	− 0.04	1
Obic1252	5	0.71	0.8	1	1	5	0.83	0.82	1	0.86	8	0.68	0.82	0.716	0.079	1
**Obic1374**	3	0.71	0.67	1	1	4	0.57	0.69	1	1	6	**0.53**	**0.72**	0.467	**0.152**	1
**Obic168**	3	**0.29**	**0.62**	0.84	1	2	0.29	0.44	1	1	6	0.63	0.76	0.467	0.064	1
Obic1	2	0.14	0.14	ND^a^	1	1	0	0	ND^a^	1	5	0.47	0.5	1	0.016	1
Obic220	2	0.57	0.53	1	1	2	0.57	0.53	1	1	3	0.53	0.5	1	− 0.049	1
**Obic415**	3	0.57	0.47	1	1	4	**0.43**	**0.71**	0.533	1	7	0.42	0.52	0.716	0.058	1
**Obic450**	2	**0.14**	**0.36**	0.94	1	2	0.29	0.44	1	1	2	0.47	0.37	0.813	− 0.134	1
Obic629	6	0.86	0.84	1	1	4	0.86	0.74	1	1	6	0.68	0.79	0.716	0.055	1
Obic73	3	0.43	0.56	1	1	3	0.71	0.7	1	1	5	0.74	0.7	1	− 0.04	1
**Obic740**	8	**0.71**	**0.90**	0.84	1	7	0.71	0.88	0.533	1	11	0.79	0.86	1	0.031	1
Obic77	3	0.43	0.39	1	1	2	0.29	0.26	1	1	3	0.32	0.28	1	− 0.081	1
Obic95	3	0.86	0.70	1	1	5	0.71	0.76	1	1	6	0.74	0.74	0.716	− 0.008	1

N_A_ = number of alleles, H_o_ = observed heterozygosity, H_E_ = expected heterozygosity, HWE = p-value for Hardy–Weinberg equilibrium test, Null = estimed null allele frequency, and P_SA_ = proportion of individuals successfully amplified. Markers in bold showed estimates of null alleles > 0.1 or disparate observed and expected heterozygosities that may be suggestive of null allleles

## Results

A single locus (Obic428) was found to be monomorphic, and was subsequently excluded from analysis. The remaining seventeen loci were in Hardy Weinberg equilibrium (p > 0.05; Table 2). Out of all 136 marker combinations (n*[n − 1]/2), for each of the three populations, none showed significant linkage disequilibrium (p > 0.05). Estimates for null allele frequencies could only be obtained for the pooled German population (n = 19), this was due to the high variability at each locus coupled with the low sample size in the English populations (n = 7, in each). Obic113 and Obic1374 showed a high estimated null allele frequency (> 0.1) for the pooled German population (Table 2). For the English populations, a large disparity between observed and expected heterozygosity (Δ > 0.2) may be indicative of the presence of null alleles, which Obic168 and Obic450 showed for the North Shropshire population (Table 2). Obic740 likewise showed a difference in observed and expected heterozygosity in this population (Table 2), albeit below 0.2 (Δ = 0.19). For the Surrey population, the expected and observed heterozygosities of Obic415 and Obic113 were also suggestive of the presence of null alleles (Table 2). Because null alleles for several markers in this study (Obic113, Obic1374, Obic168, Obic450, and Obic415) are population specific (Table 2) care must be taken when using these markers in future studies. Null alleles (as well as allelic drop out) might occur for any marker depending on the population. Therefore, estimating null allele frequency and accounting for error rates by using appropriate tests is necessary for any analysis [9].

## Discussion

Solitary bee species tend to be understudied genetically, compared to their social cousins [25]. *Osmia bicornis* is only one of two Megachilid bees to have its genome sequenced [3], and here we present seventeen newly mined and validated microsatellite markers. Additionally, the markers work in conjunction with a relatively easy extraction method [16], which lends itself to non-invasive sampling. The existing microsatellites [7] have already been used to gauge genetic differentiation in subpopulations across Europe [11]. However, even with the large sample sizes used, patterns such as isolation by distance could not be definitively inferred [11]. Power of inference relies heavily on the number and variability of loci [10], and the markers presented here are a marked improvement upon this. The markers will prove valuable in gauging the genetic diversity, inbreeding and effective population sizes of this common solitary bee. For instance, nothing is known on the impact of the species’ commercial movement on the genetic structure and health of wild populations. Furthermore, the marker set could be used to: characterize population densities and foraging range [3], help identify cryptic species [1], perform a genetic test of the mating system of the species, and study genetic diversity in relation to immunity for instance [1]. Ultimately, such studies will help inform and establish robust breeding and conservation programs [1–3]. Our new markers will supplement the six existing markers [7], bolstering the power of inference in genetic studies. The new markers have been combined and validated for use in multiplex PCR, to create a robust and powerful set of markers, suitable for cost- and time-effective genotyping.

As bee declines threaten the integrity of ecosystem function and food security [1, 3], managed and semi-managed pollinators such as *O. bicornis* and related *Osmia* species are becoming increasingly important as a supplementary pollinator force [5]—particularly for use in greenhouses and orchards. For instance, *Osmia cornifrons* and *Osmia excavata* are used in parts of Asia, where *Osmia pedicornis* and *Osmia taurus* are also being considered as managed pollinators [5]. *Osmia cornuta* is used alongside *O*. *bicornis* in Europe, and *Osmia lignaria, O. cornifrons*, and *Osmia ribifloris* are all used in the United States [5]. All of these agriculturally managed *Osmia* species (excepting *Osmia ribifloris*) belong to the ‘bicornis clade’, originating 5.6 Ma ago (4.2–7.1 Ma; [5]). Due to this close phylogenetic relationship (*O. pedicornis* and *O. taurus* having the closest relation [5]), many of the markers developed for *O. bicornis* here are likely to work in these species as well. As no other *Osmia* have had their genomes sequenced so far [3] and no microsatellite markers yet exist for these species, the microsatellite markers that are available for *Osmia bicornis* may provide a timely answer for monitoring and studying these species. Especially, considering *Osmia* species are being introduced to non-native areas due to their utility as managed pollinators, potentially driving decline in native congeners [5].

## Supplementary Information

Below is the link to the electronic supplementary material.

Supplementary file1 (XLSX 18 kb)

## Data Availability

Data is made available in the supplementary material (Online resource 1).
